# Vaccination

**Published:** 1920-11-20

**Authors:** 


### VACCINATION.

A very valuable exposition of what vaccination means to modern civilisation* has been recently published by the Research Defence Society, and although we do not wish to reopen in these pages a controversy which is as irritating to a scientific profession as it is hindering to the advancement of national health, yet we put forward below some of the more outstanding facts which the author has collected.

The present situation is aptly summarised when we realise that infant vaccination alone, as practised in this country, will not suffice, and sanitation alone will not suffice, because in actual fact the two safeguards are needed in order to provide adequate protection, and even these should be strengthened by re-vaccination at appropriate intervals. As the author points out, infant vaccination was general, although not universal, between 1840 and 1875, and yet four serious epidemics occurred during these thirty-five years, together with several minor outbreaks. On the other hand, although sanitation advances every year, and although the Government, as represented by the Ministry of Health, is increasingly anxious to prevent the importation of infection from abroad, and to deal wisely with cases when they occur, we still find smallpox as a disease so contagious and virulent that only the most watchful guardianship and drastic measures serve to keep further serious epidemics at bay.

We ourselves take it as an axiom that, from the medical officer of health's point of view, efficient public vaccination is a direct aid to organised prevention. But the people themselves have a more personal consideration. With regard to the individual, the benefit is practically unmixed, and, at least, it is very great. Whatever statistical fallacies may be arrayed against it, the medically accepted fact stands out that, setting aside for the moment certain avoidable accidents, such as may interfere with the success of any minor surgical operation, the person who has been vaccinated in infancy and re-vaccinated every ten years or so, is virtually immune from one of the most loathsome, disfiguring, and fatal of diseases. Nevertheless, more and more people seem to forget this protection. Absence or rarity of the disease appears to breed a growing contempt for its necessity, and so the community daily comes to contain an increasingly large number of unvaccinated individuals, each and all of whom are liable to infection. Among these the disease may spread like wildfire. We saw it do so in the belligerent countries during the late war—and some, at least, were more completely vaccinated than ourselves. Also we must remember that the presence of *imperfectly* vaccinated individuals among us, in whom the typical characteristics of the disease may be so masked as to deceive the medical attendant, are to be regarded as a very real danger.

The publication of the Society's review of the situation in England, and particularly the simple, straightforward fashion in which it puts forward the case of vaccination as a national necessity, should do much to localise, simplify, and make reasonable the discussions which may in future turn around the subject. The whole country is speaking enthusiastically of preventive medicine and disease control. And yet we have heard an enthusiast on this point cavil at the advisability of vaccination against smallpox. This position becomes absurd, and the sooner these things are quietly and simply explained to him the better. The little book before us is a good step in the right direction.

* "Vaccination," by Mary Scharlieb, G.B.E., M.D., M.S.; published by the Research Defence Society.

